# Toward the identification of causal genes in complex diseases: a gene-centric joint test of significance combining genomic and transcriptomic data

**DOI:** 10.1186/1753-6561-3-s7-s92

**Published:** 2009-12-15

**Authors:** Jac C Charlesworth, Juan M Peralta, Eugene Drigalenko, Harald HH Göring, Laura Almasy, Thomas D Dyer, John Blangero

**Affiliations:** 1 Department of Genetics, Southwest Foundation for Biomedical Research, P.O. Box 760549, San Antonio, Texas 78245, USA

## Abstract

**Background:**

Gene identification using linkage, association, or genome-wide expression is often underpowered. We propose that formal combination of information from multiple gene-identification approaches may lead to the identification of novel loci that are missed when only one form of information is available.

**Methods:**

Firstly, we analyze the Genetic Analysis Workshop 16 Framingham Heart Study Problem 2 genome-wide association data for HDL-cholesterol using a "gene-centric" approach. Then we formally combine the association test results with genome-wide transcriptional profiling data for high-density lipoprotein cholesterol (HDL-C), from the San Antonio Family Heart Study, using a *Z*-transform test (Stouffer's method).

**Results:**

We identified 39 genes by the joint test at a conservative 1% false-discovery rate, including 9 from the significant gene-based association test and 23 whose expression was significantly correlated with HDL-C. Seven genes identified as significant in the joint test were not independently identified by either the association or expression tests.

**Conclusion:**

This combined approach has increased power and leads to the direct nomination of novel candidate genes likely to be involved in the determination of HDL-C levels. Such information can then be used as justification for a more exhaustive search for functional sequence variation within the nominated genes. We anticipate that this type of analysis will improve our speed of identification of regulatory genes causally involved in disease risk.

## Background

The ultimate goal of genetic studies of complex diseases is the identification of the genes that are causally involved in risk. Genome-wide association studies (GWAS) (like their predecessors, linkage studies) attempt to identify genomic regions that are likely to harbor functional sequence variants influencing disease risk. For linkage studies, the size of the putative target region is on the order of 10 Mb while GWAS generally identify much smaller genomic regions of 500 kb to 1 Mb. Once such a region is found, the critical goal must then be to identify the causal gene(s) involved and their functional variants. In this paper, we propose an approach that leads to the direct nomination of empirically chosen positional candidate genes using independent transcriptional and genetic information. Once nominated, such candidate genes should be examined exhaustively to determine their causal status.

In a "successful" genome-wide association study, the result is localization of a genomic region; actual identification of causally involved genes requires substantially more information. Therefore, joint utilization of multiple sources of independent information (such as transcriptional profiling) is ultimately required to enhance inference about causal relationships. Because genes (or other contiguous genomics regions) remain the primary functional units of the human genome, we focus on gene-based tests of genetic, transcriptional, or proteomic data to determine whether a given gene is likely to be involved in the determination of a complex disease-related phenotype. This gene-centric approach allows replication studies to be focused on genes rather than variants. This analysis approach for GWAS data has been suggested as best practice [1], however it has yet to receive broad implementation.

Gene expression measurements reflect quantitative variation in transcript-specific mRNA levels and thus represent phenotypes lying very close to the direct action of genes. By globally searching for gene transcripts having levels that correlate with more classical measures of disease related phenotypes, it should be possible to nominate or prioritize novel candidate genes for more extensive genetic analysis. Combining such transcriptional information with the results of GWAS should provide a powerful approach for the selection of disease-related genes.

## Methods

As an example of our gene-centric approach, we focus on the dissection of genetic determinants influencing high-density lipoprotein cholesterol (HDL-C) levels, an important endophenotype inversely related to risk of cardiovascular disease. Age, sex, and their interactions as well as smoking status were included as covariates in all analyses.

### Framingham Heart Study data

For the genetic component of our tests, we utilized the genome-wide association information available in the Genetic Analysis Workshop 16 Problem 2 single-nucleotide polymorphism (SNP) data obtained from the long-running Framingham Heart Study (FHS) [2]. The full FHS pedigree was trimmed using PEDSYS [3] to include only genotyped individuals (*n *= 6852) plus the minimal set of untyped individuals required to maintain familial relationships. The trimmed pedigree consists of 12,789 subjects in 1059 extended families.

Genotyping was performed by Affymetrix for approximately 550,000 SNPs. The genotypes were cleaned for mendelian errors using an automated procedure in which a mistyping analysis is conducted with the computer program SimWalk2 [4,5] and those genotypes for which the probability of being mistyped exceeds a certain threshold are blanked. Genotypes were then coded as the number of copies of the minor allele. Missing genotypes were imputed (for genotyped individuals only) using the computer program Merlin [6,7].

For each cohort fasting HDL-C data were taken from the first visit for which they were available. HDL-C measures were blanked for individuals using cholesterol-lowering drugs.

### Gene-centric test of association

To quantify the evidence for a given gene influencing HDL-C levels we employed an omnibus gene-based test of association. We defined the physical location of each gene (extended by 25 kb in either direction) and then selected the Problem 2 SNPs within each region. We calculated the effective number of SNPs within a gene region using the method of Li and Ji [8] as implemented in SOLAR [9]. We then performed a marginal measured genotype analysis on each SNP using SOLAR and calculated an adjusted *p*-value for the best marginal SNP. The measured-genotype analysis [7] was conducted for each polymorphic SNP: the number of minor alleles is added to the polygenic model as a covariate in order to assess the effect of the SNP genotype on the trait mean. This model was fitted to the data and compared, using a likelihood ratio test, to the null model. Two times the difference in the log likelihoods of these models was distributed as a chi-square random variable with one degree of freedom. The likelihood ratio test statistic was recorded for each tested SNP. We adjusted the *p*-values against the effective number of SNPs using *corrected *= 1 -(1-*nominal*)^*effective*^, where *corrected *is the corrected *p*-value, *nominal *is the uncorrected *p*-value, and *effective *is the effective number of SNPs. Our approach explicitly allows for non-independence among family members and the effects of other potential covariates.

A given SNP may fall into the focal bin of more than one gene/transcript. This has the potential of inducing some positive correlation among test statistics for nearby genes. However, our reliance on the false-discovery rate (FDR) approach, with its known robustness in the presence of such positive non-independence, ameliorates this potential problem [10].

### Genome-wide transcriptional profiles

The expression analysis was conducted as part of the San Antonio Family Heart Study, initiated in 1992 to investigate the genetics of cardiovascular disease and its risk factors in Mexican Americans [11]. The expression profiling methodology is described, in detail, in Göring et al. [12]. All protocols were approved by the Institutional Review Board of the University of Texas Health Science Center at San Antonio. We used publically available information from our previously published large-scale transcriptional profiling study of lymphocyte samples from 1240 Mexican Americans [12] in which we had quantified the evidence for phenotypic correlation between HDL-C levels and gene expression levels. In this data set a χ^2 ^'tail' test was to assess whether there was a significant excess of samples with transcript-specific expression values above the 95^th ^percentile of the null distribution based on manufacturer-provided negative control samples. This allowed the detection of even those RNA molecules that are clearly present above baseline levels in some individuals. We identified a total of 22,413 transcripts with significantly detectable expression levels [12]. Using a conservative FDR of 1%, we identify 102 transcripts that were significantly correlated with HDL-C levels.

### Joint test

We then simultaneously utilized our transcriptional and genetic information by the application of a joint gene-based test that takes into account the evidence for a phenotypic relationship between HDL-C levels and a gene's expression and the strength of the association between SNPs in (or near) the gene with HDL-C levels. We combine these two tests using a *Z*-transform test also known as 'Stouffer's method" [13]. The method basically converts *p*-values to *Z*-scores using an inverse normal transformation. The *Z*-scores are summed and then scaled by the square root of the number of combined tests. The resulting test statistic is distributed as a standard normal variate that is then transformed back to a combined overall *p*-value. This omnibus test is not dependent upon the distribution of the data but depends only upon the expected uniform distribution of *p*-values under the null hypothesis.

## Results and discussion

For each cohort, the first HDL-C measure was included in the phenotype file, along with age at exam and smoking status. HDL-C measures were blanked for individuals using cholesterol-lowering drugs. In total there were 6334 individuals with both HDL-C measures and genotype data, with HDL-C measures ranging from 16 to 206 (mean 53.6 ± 0.2). Within our analysis dataset, age at exam ranged from 5 to 72 (mean 38.3 ± 0.1). There were 6301 individuals with data on both HDL-C and age and 6152 individuals with both HDL-C and smoking status.

The genes considered in this investigation were those corresponding to the 22,413 transcripts identified in the expression profiling. Of these, there were 17,350 gene regions with a least one effective SNP located within a 25-kb extension of either side of the physical gene location (NCBI build 36.3). SNP counts ranged from 1 to 597, with an average of 21 ± 1 SNPs per gene region. The 25-kb extension of the boundaries was selected to maximize the number of SNPs that may influence the target gene while minimizing the number of overlapping; this parameter is investigator-driven and can be adjusted as required.

Of the 17,350 gene regions tested, 14 were significantly associated with HDL-C from the measured genotype analysis, following correction of the *p*-value for the effective number of SNPs within the region, at a 1% FDR. These results are shown in Table 1.

**Table 1:** The 14 measured genotypes results for HDL-C significant at a 1% FDR

Gene	Chromosome	Best SNP	χ^2^	Uncorrected *p*-value	Corrected *p*-value	Number of SNPs	Effective SNPs
*CETP*	16	rs3764261	159.09	<1.0 × 10^-20^	<1.0 × 10^-20^	25	20
*HRNBP3*	17	rs898533	1621.74	<1.0 × 10^-20^	<1.0 × 10^-20^	41	36
*SMURF1*	7	rs9297145	1599.57	<1.0 × 10^-20^	<1.0 × 10^-20^	20	12
*KLHL6*	3	rs12496193	1594.56	<1.0 × 10^-20^	<1.0 × 10^-20^	32	20
*NLRC5*	16	rs11508026	121.65	<1.0 × 10^-20^	<1.0 × 10^-20^	40	31
*LPL*	8	rs17410962	52.95	3.4 × 10^-13^	4.5 × 10^-12^	20	13
*IQCG*	3	rs11547008	46.68	8.4 × 10^-12^	5.9 × 10^-11^	12	7
*ZNF613*	19	rs4987042	42.82	6.0 × 10^-11^	4.8 × 10^-10^	13	8
*RCAN2*	6	rs1442219	33.60	6.8 × 10^-9^	8.1 × 10^-8^	26	12
*CYP51A1*	7	rs2229188	29.60	5.3 × 10^-8^	2.1 × 10^-7^	9	4
*IL8*	4	rs2886920	27.90	1.3 × 10^-7^	1.5 × 10^-6^	18	12
*PACRG*	6	rs13202088	27.56	1.5 × 10^-7^	1.0 × 10^-5^	127	69
*CC2D2A*	4	rs16892095	25.32	4.9 × 10^-7^	1.1 × 10^-5^	44	23
*LYRM2*	6	rs4707557	23.98	9.7 × 10^-7^	9.7 × 10^-6^	13	10

In the joint test there were a total of 39 genes significant at a highly conservative 1% FDR, including 9 from the significant measured genotype set and 23 with expression that was significantly correlated with HDL-C. Seven genes identified as significant in the joint test were not identified by either the association or expression tests independently (*ABCG1*, *C12orf62*, *C6orf64*, *GPBAR1*, *LOC283551*, *LYRM1*, and *PRPF38A*). The results of the joint test are shown in Additional File 1.

The genes shown in Table 2 are prime candidates for resequencing and variant typing, empirically selected based on evidence both from transcriptional profiling and genome-wide association. One of the most significant genes is *CETP *(cholesteryl ester transfer protein), a well known cholesterol binding gene. In total, there are seven well known lipid metabolism genes prioritized by the joint test (*ABCB4*, *ABCG1*, *CETP*, *CYP51A1*, *IL8*, *IL1R2*, and *LPL*). Interestingly, the list also prioritizes a number of genes of little-known function, such as *NLRC5 *(NLR family CARD domain containing 5), *TCTN1 *(tectonic family member 1), and *TPPP3 *(tubulin polymerization-promoting protein family member 3), which would not be selected by any form of candidate gene approach.

It can also be seen that there are situations in which genes show a highly significant correlation between their expression and HDL-C, but no evidence of association at the physical location of the gene, such as *IL1R2 *(Table 2). Similarly, there are cases (*SMURF1*) where the association information drives the combined tests. We have retained all genes that exhibit combined significance. An individual reader may choose to further focus on only those genes that exhibit at least nominal significance on each dimension.

While this approach shows great potential for speeding gene identification, it also has several limitations. One potential weakness is the focus on regulatory variation. While there is a growing belief that much of quantitative phenotypic variation may stem from regulatory variation, other types of mechanisms (e.g., structural variation that alters protein-protein interactions) can also be involved. Similarly, genes whose expression is not detected in the target tissue may be missed. Thus, as with all discovery-based approaches, only positive findings admit interpretation. A gene cannot be ruled out using these methods.

This paper combines information from two different population studies. Both samples, however, are ascertained without regard to phenotype. It is possible that the relationship between expression levels and disease-related phenotypes may vary across populations. However, we would expect this to diminish signal rather than yield false positives. Optimally, expression and association results would come from the same data set.

## Conclusion

Our results suggest that the formal combination of information from orthogonal sources may lead to the identification of novel loci that are missed when only one form of information is available. For the current study, we have combined existing information on the correlation of gene expression levels with HDL-C and the association between SNPs near these genes with HDL-C levels. Our simple measure of evidence is effectively a measure of significance resulting from the combination of *p*-values from two separate tests, both of which are tests of a gene-centric hypothesis. Of course, this approach can be made substantially more powerful when both forms of information are available in a single study and a formal, true joint test is specified. In the current application, our results empirically nominate genes that are likely to be directly involved in quantitative HDL-C variation. Many of these genes would *not *have been identified using a classical pathways-based combinatorial approach because their functions have yet to be identified. Many would also not have been identified by using each approach in isolation. A logical next step would be either replication or, given the magnitude of current evidence, a direct move to resequencing to identify functional variants.

## List of abbreviations used

FDR: False-discovery rate; FHS: Framingham Heart Study; GAW16: Genetic Analysis Workshop 16; GWAS: Genome-wide association study; HDL-C: High-density lipoprotein cholesterol; SNP: Single-nucleotide polymorphism.

## Competing interests

The authors declare that they have no competing interests.

## Authors' contributions

JCC and JB wrote the paper. JCC, JMP, ED, HHHG, and TDD processed and analyzed the data. JB conceived the methodology and fundamental structure of the project. JCC, HHHG, and LA supported the conception and design of the project.

## Supplementary Material

Additional file 1 The 39 significant joint-test results for HDL-C significant at a 1% FDR. Click here for file
