# Supplementary material for: Toward the identification of causal genes in complex diseases: a gene-centric joint test of significance combining genomic and transcriptomic data
Source: BMC Proc. 2009 Dec 15;3(Suppl 7):S92. doi: 10.1186/1753-6561-3-s7-s92 (PMC2795996; doi:10.1186/1753-6561-3-s7-s92)
Supplement: Additional file 1 — The 39 significant joint-test results for HDL-C significant at a 1% FDR. [file 1753-6561-3-S7-S92-S1.pdf]

**Supplemental File 1 - The 39 significant joint-test results for HDL-C significant at a 1% FDR**

| Gene | Illumina transcript ID | Chromosome | Association *p*-value | Expression *p*-value | Multi-*Z* *p*-value |
|---|---|---|---|---|---|
| *CETP* | GI_4557442-S | 16 | $<1.0\times10^{-20}$ | $2.4\times10^{-1}$ | $<1.0\times10^{-20}$ |
| *SMURF1* | GI_31317289-A | 7 | $<1.0\times10^{-20}$ | $7.1\times10^{-1}$ | $<1.0\times10^{-20}$ |
| *KLHL6* | GI_18482382-S | 3 | $<1.0\times10^{-20}$ | $2.2\times10^{-1}$ | $<1.0\times10^{-20}$ |
| *NLRC5* | GI_28951070-S | 16 | $<1.0\times10^{-20}$ | $3.3\times10^{-1}$ | $<1.0\times10^{-20}$ |
| *HRNBP3* | hmm23957-S | 17 | $<1.0\times10^{-20}$ | $3.5\times10^{-1}$ | $<1.0\times10^{-20}$ |
| *LPL* | GI_4557726-S | 8 | $4.5\times10^{-12}$ | $4.8\times10^{-4}$ | $4.1\times10^{-13}$ |
| *TCTN1* | GI_31542721-S | 12 | $2.3\times10^{-3}$ | $3.0\times10^{-8}$ | $2.6\times10^{-9}$ |
| *TRAK2* | GI_13027379-S | 2 | $6.3\times10^{-4}$ | $1.2\times10^{-5}$ | $6.8\times10^{-8}$ |
| *IL1R2* | GI_27894333-A | 2 | $2.8\times10^{-1}$ | $<1.0\times10^{-20}$ | $1.2\times10^{-7}$ |
| *IQCG* | GI_31340578-S | 3 | $5.9\times10^{-11}$ | $2.0\times10^{-1}$ | $1.2\times10^{-7}$ |
| *FLJ43505* | GI_42656096-S | 1 | $1.7\times10^{-1}$ | $4.0\times10^{-10}$ | $2.5\times10^{-7}$ |
| *TPPP3* | GI_7706275-A | 16 | $1.7\times10^{-3}$ | $1.8\times10^{-5}$ | $3.0\times10^{-7}$ |
| *ZNF613* | GI_21362002-S | 19 | $4.8\times10^{-10}$ | $1.9\times10^{-1}$ | $3.8\times10^{-7}$ |
| *CDCA7L* | GI_31542536-S | 7 | $1.2\times10^{-1}$ | $7.6\times10^{-9}$ | $6.8\times10^{-7}$ |
| *ZDHHC23* | GI_27734844-S | 3 | $2.0\times10^{-1}$ | $2.1\times10^{-9}$ | $1.0\times10^{-6}$ |
| *RTN1* | GI_10863934-S | 14 | $9.2\times10^{-3}$ | $9.8\times10^{-6}$ | $1.4\times10^{-6}$ |
| *SLC6A16* | GI_7662517-S | 19 | $4.6\times10^{-2}$ | $4.5\times10^{-7}$ | $1.5\times10^{-6}$ |
| *C12orf62* | GI_34147446-S | 12 | $1.9\times10^{-3}$ | $1.0\times10^{-4}$ | $1.5\times10^{-6}$ |

| | | | | | |
|---|---|---|---|---|---|
| *ABCB4* | GI_9961251-A | 7 | $1.7\times10^{-1}$ | $1.0\times10^{-8}$ | $1.8\times10^{-6}$ |
| *MGC52498* | hmm8270-S | 1 | $4.1\times10^{-2}$ | $2.0\times10^{-6}$ | $3.5\times10^{-6}$ |
| *C12orf57* | GI_34147536-S | 12 | $1.8\times10^{-2}$ | $2.3\times10^{-5}$ | $6.4\times10^{-6}$ |
| *NUP88* | GI_24497452-S | 17 | $9.2\times10^{-2}$ | $6.4\times10^{-7}$ | $6.5\times10^{-6}$ |
| *IL4R* | GI_4557668-S | 16 | $2.4\times10^{-1}$ | $2.9\times10^{-8}$ | $7.1\times10^{-6}$ |
| *ABCG1* | GI_8051576-A | 21 | $2.8\times10^{-3}$ | $4.7\times10^{-4}$ | $8.6\times10^{-6}$ |
| *LYRM1* | GI_19923825-S | 16 | $7.8\times10^{-4}$ | $1.8\times10^{-3}$ | $8.7\times10^{-6}$ |
| *NASP* | GI_27262629-A | 1 | $2.4\times10^{-2}$ | $2.3\times10^{-5}$ | $9.4\times10^{-6}$ |
| *PKIG* | GI_32483384-A | 20 | $1.5\times10^{-1}$ | $2.8\times10^{-7}$ | $1.0\times10^{-5}$ |
| *PPAPDC1B* | GI_40316920-S | 8 | $1.7\times10^{-1}$ | $2.6\times10^{-7}$ | $1.2\times10^{-5}$ |
| *LOC283663* | GI_42660461-S | 15 | $2.9\times10^{-1}$ | $4.3\times10^{-8}$ | $1.5\times10^{-5}$ |
| *FAM129C* | GI_31542206-S | 19 | $3.6\times10^{-2}$ | $2.2\times10^{-5}$ | $1.6\times10^{-5}$ |
| *LILRB2* | GI_5031910-S | 19 | $2.6\times10^{-1}$ | $8.7\times10^{-8}$ | $1.7\times10^{-5}$ |
| *MAPK7* | GI_20986498-A | 17 | $2.5\times10^{-2}$ | $5.2\times10^{-5}$ | $1.8\times10^{-5}$ |
| *C6orf64* | GI_8922866-S | 6 | $4.7\times10^{-4}$ | $6.6\times10^{-3}$ | $2.1\times10^{-5}$ |
| *LOC283551* | GI_27479394-S | 14 | $1.7\times10^{-2}$ | $1.3\times10^{-4}$ | $2.3\times10^{-5}$ |
| *CYP51A1* | GI_13929427-S | 7 | $2.1\times10^{-7}$ | $2.4\times10^{-1}$ | $2.4\times10^{-5}$ |
| *USP7* | GI_4507856-S | 16 | $4.4\times10^{-2}$ | $2.8\times10^{-5}$ | $2.5\times10^{-5}$ |
| *PRPF38A* | GI_24762235-I | 1 | $1.0\times10^{-2}$ | $3.2\times10^{-4}$ | $2.6\times10^{-5}$ |
| *C22orf32* | GI_34222144-S | 22 | $1.1\times10^{-1}$ | $4.1\times10^{-6}$ | $2.7\times10^{-5}$ |
| *GPBAR1* | GI_24850108-S | 2 | $1.1\times10^{-3}$ | $4.1\times10^{-3}$ | $2.7\times10^{-5}$ |
